# Successful management of peripartum cardiomyopathy in a Kenyan setting: a case series

**DOI:** 10.11604/pamj.2023.44.150.38455

**Published:** 2023-03-28

**Authors:** Khushboo Jayant Sonigra, Esther Nyambura, Oscar Mwangi, Krishan Sarna, Kireki Omanwa

**Affiliations:** 1 Department of Obstetrics and Gynecology, University of Nairobi, Nairobi, Kenya,; 2 Department of Dental Sciences, Unit of Oral and Maxillofacial Surgery, Oral Pathology and Oral Medicine, University of Nairobi, Nairobi, Kenya

**Keywords:** Peripartum cardiomyopathy, postpartum cardiomyopathy, heart failure, multidisciplinary approach, management

## Abstract

Peripartum cardiomyopathy is a rare life-threatening condition occurring in previously healthy women with symptoms mimicking those of normal pregnancy and is associated with a high mortality rate. A high index of suspicion coupled with a sound understanding of the disease is crucial to correctly diagnose and manage the patients to improve final maternal outcomes. In this report, we present a total of five cases of peripartum cardiomyopathy in women aged 22 to 38 years who presented between 3 and 21 days postpartum. All patients presented with severely reduced ejection fractions indicative of heart failure and were immediately admitted to our facility. A timely diagnosis was made and patients started on a combination of antibiotics, anticoagulants, and anti-heart failure medication. Despite the severity of the disease upon presentation, early diagnosis and precise management of the disease were essential in achieving favorable patient outcomes. Therefore, this report provides crucial knowledge about the presentation and progression of peripartum cardiomyopathy and presents a treatment protocol from a Kenyan perspective that was successfully employed in the management of all five cases.

## Introduction

Peripartum cardiomyopathy (PPCM) is a rare, and potentially fatal form of dilated cardiomyopathy (DCM) occurring in the peripartum period. Its prevalence is 1 in 1300 to 1 in 5,000 pregnancies and is more common in developing countries [1]. The four main diagnostic criteria of PPCM are captured within its definition: i) an idiopathic cardiomyopathy; ii) occurring in the final month of pregnancy or within five months of delivery; iii) presenting with heart failure secondary to left ventricular systolic dysfunction thereby; iv) reducing the left ventricular ejection fraction (LVEF) to less than 45% or fractional shortening to less than 30% [2]. The precise etiopathophysiology of PPCM remains unknown, however, it is likely to be multifactorial and may involve combinations of autoimmune conditions, nutritional deficiencies, genetic causes, viral infections, or maybe truly idiopathic. Increased incidences of PPCM have been observed in women with pre-eclampsia, chronic hypertension, smoking, alcoholism, multiparty, advanced maternal age, multiple pregnancies, malnutrition, and long-term tocolysis, and is more common among those of African descent [3]. The clinical characteristics of PPCM include dyspnea, dizziness, orthopnea, fatigue, lower limb edema, tachycardia, palpitations, productive cough, and abdominal tenderness [4]. This makes the diagnosis of this condition quite challenging considering that some of these symptoms are quite common in the normal range of peripartum circumstances and may be disguised as such, therefore, evading a possible diagnosis of PPCM. Untreated PPCM greatly elevates the risk of complications such as cardiogenic shock, arrhythmias, heart failure, cardiopulmonary arrest, and death [4]. It is therefore critical to have a high index of suspicion following a new or rapid onset of symptoms towards the end of pregnancy or after delivery bearing in mind that an early diagnosis has significantly better maternal outcomes [5]. Management of PPCM should consist of a multidisciplinary approach involving gynecologists, cardiologists, intensive care unit specialists, and cardiac surgeons to improve outcomes [1]. In this paper, we report five African patients presenting with PPCM who were managed successfully through prompt diagnosis, early treatment, and comprehensive follow-ups. Furthermore, we review the etiology, clinical symptoms, and management of PPCM of which clinicians must have a sound understanding delivering efficient and appropriate care.

## Methods

All five patients in this case series presented to the Kenyatta National Teaching and Referral Hospital within a span of two weeks with signs and symptoms suggestive of PPCM. The hospital is one of the largest public tertiary referral centers in Kenya and Eastern Africa where each patient is managed by a multidisciplinary team of specialists thus allowing improved patient care and outcome. Both the infrastructure and resources available for the diagnosis and management of such conditions conform to internationally accepted standards. Ethical committee approval for this paper was not required, however, both verbal and written consent was obtained from each patient for the publishing of this report. The data collected that was of interest specific to these particular cases included: the patient´s presenting complaint, past medical history, obstetric history, examination findings (general and systemic), findings from laboratory and radiological investigations, comprehensive treatment plan, and patient outcomes. The data was organized, tabulated, and synthesized before presentation in this report. This retrospective case series conformed to the Declaration of Helsinki.

## Results

**Figure 1:**
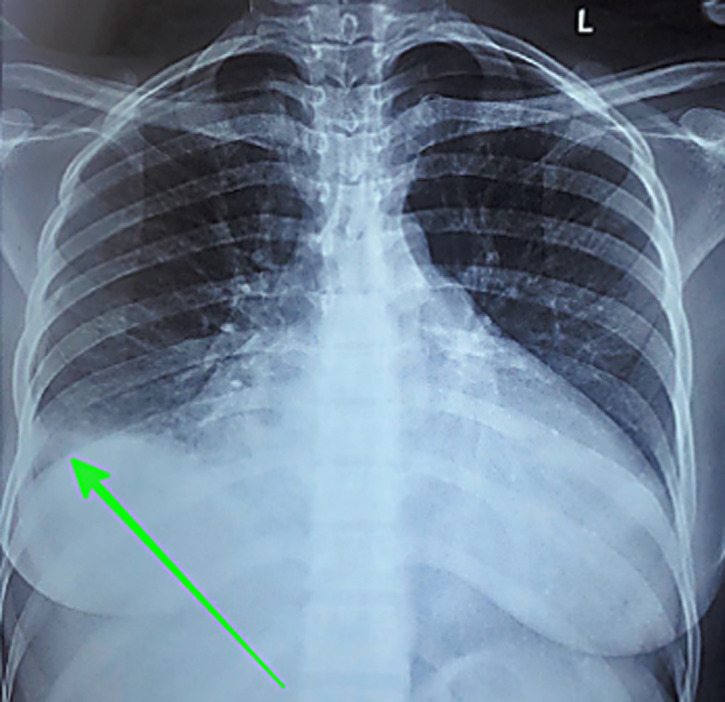
chest X-ray showing a right pleural effusion (green arrow)

**Case 1:** a 38-year-old African female, para 3+0, gave birth to a healthy male newborn weighing 3,010 g through an emergency cesarean section (EMCS) (Table 1). After a period of five days, she developed difficulty in breathing (New York Heart Association (NYHA) class IV) and excessive productive cough with hemoptysis. Past medical history was non-contributory. Upon examination, the patient appeared to be sick looking in severe respiratory distress with a respiratory rate of 30 breaths per minute. Blood pressure was found to be 125/81 mm Hg, however, the patient had tachycardia of 125 beats per minute and oxygen saturation of 79% on room air and 94% on oxygen. Furthermore, the presence of lower limb edema was noted accompanied by jugular venous distention, S3 gallop, and hepatomegaly of 4 cm below the costal margin. Bilateral crepitations were also heard on respiratory examination. The laboratory investigations on admission revealed a normal hematological profile apart from an elevated white blood cell count of 11.84x10^9^/L. COVID-19 and sputum gene X-pert tests for tuberculosis were negative. An elevated D-dimer level was found of 1595 ug/dl. Radiological investigations including a plain chest X-ray, electrocardiograph, echocardiogram (Figure 1), and computed tomography (CT) pulmonary angiogram were performed whose findings have been summarized in Table 2 below. Of note, the patient was found to have bilateral pleural effusion and a left ventricular apical thrombus. After careful interpretation of all the results, the patient was diagnosed with new-onset PPCM in cardiac failure and was immediately admitted to the intensive care unit (ICU). Here, the patient was intubated and started on the medications summarized in Table 3. Nebulization was done every 4 hours and chest physiotherapy was initiated. On day five of admission, the patient´s condition improved and was transferred to the acute gynecology ward on oxygen. The patient was discharged on day 20 after admission with the same medications through the cardiac clinic. She remains well to date with no further complications reported.

**Table 1:** patient demographic information for all five cases

Demographics	Case 1	Case 2	Case 3	Case 4	Case 5
Maternal age (years)	38	22	19	34	25
Gestational age (weeks)	38	35	39	40	40
Clinical history	None	Preeclampsia	None	None	None
Previous pregnancies	2, normal	0	0	2, normal	0
Type of delivery	Cesarean	Vaginal	Vaginal	Cesarean	vaginal
Tocolytic therapy	No	No	No	No	No
Complications of delivery	None	Hypertension	None	None	None
Fetus	Normal	Intrauterine fetal demise	Normal	Normal	Normal
Moment of clinical onset	5 days post delivery	11 days post delivery	3 days post delivery	15 days post delivery	21 days post delivery
Clinical debut	Heart failure	Heart failure	Heart failure	Heart failure	Heart failure
Length of stay in hospital	20 days (5 days in ICU^1^and 15 days in the ward)	6 days in the ward	8 days in the ward	7 days in the ward	5 days in the ward

**Table 2:** a summary of the radiological investigations performed and the findings of each

Investigation	Case 1	Case 2	Case 3	Case 4	Case 5
Chest radiography	Cardiomegaly with bilateral lung opacifications with bilateral pleural effusion	Cardiomegaly	Cardiomegaly with bilateral pleural effusion	Cardiomegaly with unilateral pleural effusion	Cardiomegaly with bilateral mild pleural effusions
Electrocardiograph	Sinus tachycardia with ST elevation	Sinus tachycardia with left atrial enlargement with ST elevation	Sinus tachycardia with ST elevation	Normal sinus rhythm	Sinus tachycardia
Echocardiogram	DCM [1] with severe biventricular systolic and diastolic dysfunction. -Left ventricular ejection fraction of 24.2%. -Moderate mitral regurgitation. -Dilated chamber with global hypokinesia. -Left ventricular apical thrombus.	-DCM with severe left ventricular systolic dysfunction. -Left ventricular ejection fraction of 17%. -Moderate mitral regurgitation. -Dilated left ventricle with global hypokinesia.	-DCM with severe biventricular systolic and diastolic dysfunction. - Left ventricular ejection fraction of 23%. - Moderate mitral and tricuspid regurgitation and pulmonary arterial hypertension	- DCM with severe biventricular systolic and diastolic dysfunction. -Left ventricular ejection fraction of 27%. -Moderate mitral regurgitation. -Dilated chamber with global hypokinesia. -Small rim of pericardial effusion.	-DCM with severe biventricular systolic and diastolic dysfunction. - Left ventricular ejection fraction of 22%. -Moderate tricuspid regurgitation with mild pulmonary atrial hypertension and severe mitral regurgitation. - Mild pleural effusion with global hypokinesia.
Computed tomography pulmonary angiogram	Bilateral pleural effusion with bilateral ground glass opacifications suggestive of pulmonary edema and no evidence of pulmonary emboli	No evidence of pulmonary emboli	Bilateral pleural effusion with pericardial effusion and no evidence of pulmonary emboli	No evidence for pulmonary emboli	No evidence of pulmonary emboli

**Table 3:** a summary of the medications used in the management of each case

Treatment	Case 1	Case 2	Case 3	Case 4	Case 5
**Medications**	IV^1^ Furosemide 60mg twice daily	PO Aldomet 500mg once daily	IV Furosemide 60mg thrice daily	IV Furosemide 60mg thrice daily	IV Furosemide 60mg thrice daily
	PO^1^ Aldactone 25mg once daily		PO Aldactone 12.5mg once daily		
	PO Enalapril 2.5mg twice daily	IV Furosemide 80mg once daily	PO Enalapril 2.5mg twice daily	PO Carvedilol 3.125mg twice daily	PO Digoxin 0.125mg once daily
	PO Digoxin 0.125mg once daily	PO Digoxin 0.25mg once daily	PO Digoxin 0.125mg once daily	PO Enalapril 2.5mg once daily	PO Carvedilol 6.25mg twice daily
	SC^1^ Clexane 80mg once daily	PO Aldactone 50mg once daily	PO Bromocriptine 2.5mg once daily	SC Clexane 80mg once daily	PO Empagliflozin 10mg once daily
	PO Esomeprazole 40mg twice daily	SC Clexane 60mg once daily	SC Clexane 40mg once daily	PO Bromocriptine 2.5mg once daily	SC Clexane 60mg once daily
	PO Bromocriptine 2.5mg once daily	PO carbegoline 1mg once daily for 2 days	PO Ivabradine 5mg once daily	Ranferon 10mls thrice daily for a month	PO Bromocriptine 2.5mg once daily
**Antibiotics**	IV Ceftriaxone 1g twice daily for 10 days	IV Augmentin 1g twice daily for 5 days	IV Ceftriaxone 1g twice daily for 5 days	IV Ceftriaxone 1g twice daily for 5 days	IV Augmentin 1g twice daily for 5 days
	IV Azithromycin 500mg once daily for 3 days	IV Metronidazole 500mg twice daily for 5 days	IV Clarithromycin 500mg twice daily for 5 days		IV Metronidazole 500mg twice daily for 5 days
**Outcome**	Patient improved clinically - achieved full recovery				
	Discharged on the same medications through the cardiac clinic for follow-up for 6 months -1 year				
	Clexane substituted with PO rivaroxaban 15mg twice daily for the first three weeks				

**Figure 2:**
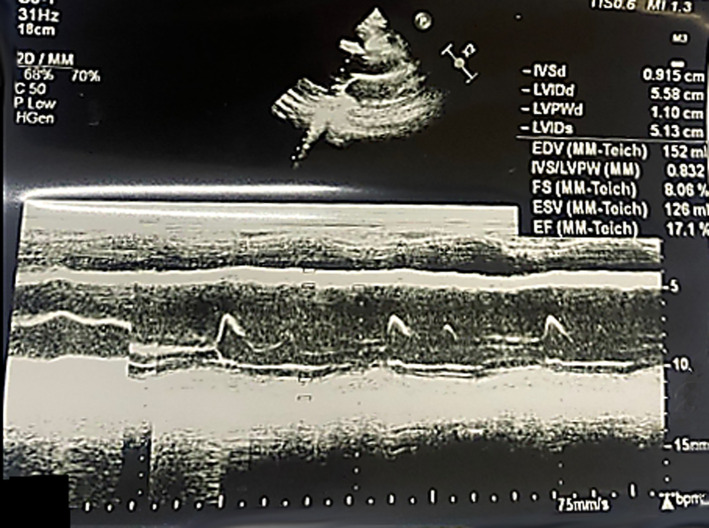
transthoracic echocardiography in long-axis view showing left ventricular ejection fraction of 17.1%

**Case 2:** a 22-year-old African female, para 0+1, in her third trimester of pregnancy (35 weeks of gestation) presented with an intrauterine fetal demise of a twin gestation due to twin-to-twin transfusion syndrome. Induction of labor was performed to expel the fetuses and the patient was consequently discharged in good health. After a period of 11 days, the patient returned to the emergency department with difficulty in breathing (NYHA class IV), excessive productive cough, easy fatiguability, and bilateral lower limb edema. She reported having paroxysmal nocturnal dyspnea and palpitations. Past medical history was non-contributory except for the complications during pregnancy mentioned. The patient appeared in obvious respiratory distress with a high blood pressure of 149/78 mm Hg, a tachycardia of 118 beats per minute, a respiratory rate of 30 breaths per minute, and oxygen saturation of 80% on room air and 89% on 2 liters/min of oxygen. Physical examination showed bilateral lower limb pitting edema, jugular venous distention, and systolic murmur. On respiratory exam, bilateral fine crepitations were heard. Laboratory investigations on admission revealed normal hematological levels. The COVID-19 test was negative and D-dimer levels were within range. A chest X-ray, an electrocardiograph, an echocardiogram, and a computed tomography pulmonary angiogram were performed (Table 2). Of note, the patient had a very low ejection fraction of 17.1% (Figure 2). The patient was admitted to the ward for new-onset PPCM in cardiac failure and was started on the medications summarized in Table 3. On day four of admission, the patient improved clinically and was discharged. She remains well to date with no complications reported to date.

**Case 3:** a 22-year-old African female, para 1+0, presented to the emergency department three days after the spontaneous vaginal delivery of a healthy female newborn weighing 2,870g. The patient reported dyspnea, easy fatiguability, palpitations, and a productive cough that started two days prior in the peripheral facility where she gave birth (Table 1). Furthermore, during this time, she had two attacks of breathlessness, chest pains, and tachycardia where she was resuscitated with oxygen, and intravenous fluids, and received 1 unit of blood. She was then referred to our facility for detailed workup and management. Upon examination, she was found to be afebrile, dyspneic, orthopneic, and hypoxic with oxygen saturations of 68% on room air and was immediately put on oxygen. She was tachycardic with a pulse rate of 140 beats per minute and tachypneic with a respiratory rate of 31 breaths per minute along with a blood pressure of 127/96 mmHg. Furthermore, that tender hepatomegaly (4cm below the costal margin) was noted. Her right lower lung lobe had reduced air entry upon auscultation of the lungs. Her past medical history was non-contributory. Laboratory investigations on admission revealed normal hematological levels apart from an elevated white blood cell count of 22.41x10^9^/L. The COVID-19 test was negative. The D-dimer levels were within normal ranges. A chest X-ray, an electrocardiograph, an echocardiogram, and a computed tomography pulmonary angiogram were performed (Table 2). After careful interpretation of the results, the patient was subsequently diagnosed with new-onset PPCM in cardiac failure and was started on the medications summarized in Table 3. On day eight of admission, the patient has been weaned off oxygen and discharged. She remains well to date with no further complications reported.

**Case 4:** a 34-year-old African female, para 3+0, who delivered a healthy female newborn weighing 2,790g, presented to the emergency department 15 days post elective Cesarean section with difficulty in breathing (NYHA class IV), excessive productive cough with hemoptysis and bilateral lower limb swelling until the ankles. She reported having orthopnea, paroxysmal nocturnal dyspnea, and palpitations. Her past medical history was unremarkable. Upon presentation, the patient was in severe respiratory distress on 10L/minute of oxygen via a non-rebreather mask. She was afebrile with a blood pressure of 150/99 mm Hg, a pulse rate of 55 beats per minute, a respiratory rate of 21 breaths per minute, and an oxygen saturation of 85% on room air and 98% on oxygen. Physical examination showed bilateral lower limb pitting edema, jugular venous distention, and an S3 gallop. On respiratory exam, crepitations were heard on the right side with reduced air entry on the same side. Laboratory investigations on admission revealed normal hematological levels apart from an elevated white blood cell count of 16.24x10^9^/L and an elevated C-reactive protein of 83. COVID-19 and sputum gene X-pert tests for tuberculosis were negative. A Doppler ultrasound for both limbs was normal. D-dimer levels were within normal. A chest X-ray, an electrocardiograph, an echocardiogram, and a computed tomography pulmonary angiogram were performed (Table 2). Of note, the latter revealed a unilateral pleural effusion on the right side. The patient was admitted to the ward for new-onset PPCM in cardiac failure and was started on the medications summarized in Table 3. On day seven of admission, the patient was doing well and was weaned off oxygen. Her cardiac function improved over a period of one week and her ejection fraction had improved to 58% and was discharged. She remains well to date.

**Case 5:** a 25-year-old African female, para 1+0, who delivered a healthy female newborn weighing 2,600g, presented to the emergency department 21 days post spontaneous vaginal delivery with difficulty in breathing (NYHA class II), excessive productive cough, easy fatigability, and bilateral lower limb swelling until the ankles. She reported having paroxysmal nocturnal dyspnea and palpitations. Her past medical history was unremarkable. In the emergency department, the patient appeared in obvious respiratory distress. She was afebrile with a blood pressure of 99/60 mm Hg, a pulse rate of 109 beats per minute, a respiratory rate of 23 breaths per minute, and an oxygen saturation of 91% on room air. Physical examination showed bilateral lower limb pitting edema, jugular venous distention, and an S3 gallop and systolic mitral murmur. On respiratory exam, stony dullness on both lung bases and bilateral coarse crepitations were heard. Laboratory investigations on admission revealed normal hematological levels. The COVID-19 test was negative. D-dimer levels were within normal ranges. A chest X-ray, an electrocardiograph, an echocardiogram, and a computed tomography pulmonary angiogram were done (Table 2). The patient was admitted to the ward for new-onset PPCM in cardiac failure and was started on the medications summarized in Table 3. On day five of admission, the patient improved clinically and was discharged. She remains well to date.

## Discussion

Peripartum cardiomyopathy is a life-threatening condition affecting thousands of women worldwide and has deadly consequences if misdiagnosed or left untreated [1]. Yet, the cause of PPCM is still not fully understood. Increased maternal age, parity, hypertension, and African ethnicity have been associated with increased incidences of PPCM, however, the cases described in this report diverge from the previously postulated understanding of these factors [6]. Literature reveals that PPCM is most commonly diagnosed at the age of 30 years and older, however, in the present report, cases 2, 3, and 5 were in fact below this age [7]. Additionally, it is reported that multiparous women are also more likely to develop PPCM due to increased gestational weight leading to volume expansion and an increase in the risk of cardiac overload and strain on the heart. This stands in contrast to our case series where cases 2, 3, and 5 were in fact nulliparous [7]. Despite these dissimilarities, our report is similar to that published in India which revealed that most of the patients who developed PPCM were young primigravidae [7]. The majority of patients with PPCM also have an underlying diagnosis of chronic hypertension and preeclampsia which can make the diagnosis and management of PPCM more difficult which was the scenario observed in case 2 of this series. A study by Goli *et al*. 2021 further revealed that a genetic analysis of PPCM patients showed high frequencies of truncating variants in the TTN (TTNtvs) gene which are also high in frequency in DCM patients [8]. Hence, some patients might have a predisposition to genetic PPCM [8]. It is worth mentioning that this may have been unlikely in the patients within this case series owing to the fact that there was no family history of PPCM in all cases that were managed. Taking into consideration all the differences observed between our cases and those published in international literature, it is important to understand that the clinical characteristics of PPCM are not cast in stone and may vary between patients.

Peripartum cardiomyopathy has been postulated to be a disease of vascular etiology that is triggered by the hormonal changes of late pregnancy [9]. The peripartum period causes the pituitary and placenta to secrete hormones such as prolactin and sFlt1. Prolactin is degraded to 16-kDa vasoinhibin which then causes endothelial cell apoptosis, as well as the secretion of miRNA146a, encapsulated in exosomes. Cardiomyocytes internalize these exosomes, causing dysfunction and apoptosis. SFlt1 binds to and inhibits VEGF signaling, causing endothelial cell dysfunction and apoptosis. Finally, decreased vascular support causes metabolic insufficiency in cardiomyocytes, resulting in cardiomyopathy [10,11]. Also, there is speculation that PPCM stems from the coincidence of two “hits”: one hit is the late-gestational vasculotoxic hormonal environment, including sFlt1 and prolactin, and the second hit is an inability, in some women, to withstand this vasculotoxic insult [8,12]. Other hypotheses have been proposed including viral myocarditis, abnormal immune response, abnormal hemodynamic response, apoptosis and inflammation, cytokine imbalance, selenium deficiencies, malnutrition, and prolonged tocolysis [13]. In the cases discussed within this report, the most likely causative mechanism of PPCM appears to be related to the hormonal hypothesis as described above, as there was no additional clinical evidence that supported any other mechanism. It takes a high index of suspicion to spot the early signs of heart failure to initiate prompt diagnosis and treatment, especially in PPCM due to the similarities in clinical presentation to that of normal pregnancy [8]. The diagnosis of PPCM in all the cases presented in our report is based on a combination of the clinical picture of the patient that includes signs and symptoms of congestive heart failure and the findings of the echocardiogram of a depressed fractional shortening and ejection fraction during and immediately after birth. All of the ECHO findings in our case series in puerperal Kenyan women indicated a left ventricular ejection fraction of 17-27%, which is similar to numerous reports worldwide, some of which include the USA, China, South Africa, and India [12,14-16].

High rates of maternal and neonatal morbidity and mortality are associated with PPCM [17]. It is vital to approach such patients with a multidisciplinary team to provide appropriate care for both the mother and fetus. Prompt referral by the gynecologist to the cardiologist is crucial to improve the prognosis of the patient. According to published literature, the treatment of PPCM is similar to that used during the management of congestive heart failure. In line with these protocols, a similar regimen was employed in all our cases. Furthermore, bromocriptine, a dopamine receptor agonist, was also administered hence reducing prolactin production by eliminating the cleaved form of prolactin despite the activation of the cleaving enzyme [18]. Furthermore, patients with PPCM are at high risk of thrombus formation and therefore anticoagulation therapy must be initiated [19]. If all medical treatment fails, the last resort is heart transplantation [20]. After comprehensive management of our cases, all 5 cases recovered to great extent evidenced by improvement of ejection fractions on follow-up echocardiograms and the resolution of accompanying symptoms. Peripartum cardiomyopathy recurs in more than 30% of future pregnancies, putting both mother and baby at risk, hence it is typically advised that these patients not be pregnant again [5]. Considering that this report presents a total of five cases, it may be advantageous to conduct prospective studies with a larger sample size to conclusively establish the information provided by this manuscript.

## Conclusion

Peripartum cardiomyopathy is a life-threatening condition that substantially affects cardiovascular health and has long-lasting implications for future pregnancies. The cases presented in this report show the diverse nature of the clinical presentation of PPCM which makes it clear that vigilance is required on the part of the clinician to make an accurate and timely diagnosis of the condition. Indeed, this is a major determinant of final maternal outcomes. Furthermore, the diagnostic procedures and management protocol have been presented which allowed the successful management of PPCM in our Kenyan setting.

### What is known about this topic

- *Peripartum cardiomyopathy has a high mortality rate of between 18% to 56% worldwide;*
- *The diagnosis can be extremely challenging due to similarities to normal pregnancy;*
- *A delayed diagnosis and management of the condition may have a detrimental outcome for the patient involved*.

### What this study adds

- *Heightened vigilance is required in PPCM to ensure an accurate and timely diagnosis;*
- *This study provides a systematic diagnostic approach and management protocol for PPCM which can be used by other clinicians;*
- *A multidisciplinary approach in the management of PPCM must be employed to improve patient outcomes*.
